# Identification of *HMGA2* as a predictive biomarker of response to bintrafusp alfa in a phase 1 trial in patients with advanced triple-negative breast cancer

**DOI:** 10.3389/fonc.2022.981940

**Published:** 2022-12-08

**Authors:** Alexander Spira, Ahmad Awada, Nicolas Isambert, David Lorente, Nicolas Penel, Yue Zhang, Laureen S. Ojalvo, Christine Hicking, P. Alexander Rolfe, Christian Ihling, Isabelle Dussault, George Locke, Christian Borel

**Affiliations:** ^1^ Department of Medical Oncology, Virginia Cancer Specialists, Fairfax, VA, United States; ^2^ US Oncology Research, The Woodlands, TX, United States; ^3^ Medical Oncology Clinic, Institut Jules Bordet, Université Libre de Bruxelles, Brussels, Belgium; ^4^ Department of Medical Oncology, Centre Georges-François Leclerc, Dijon, France; ^5^ Department of Medical Oncology, Hospital Universitari I Politècnic La Fe, Valencia, Spain; ^6^ Department of Medical Oncology, Centre Oscar Lambret, Lille, France; ^7^ Department of Medical Oncology, Université de Lille, Lille, France; ^8^ EMD Serono Research & Development Institute, Inc, an Affiliate of Merck KGaA, Billerica, MA, United States; ^9^ Merck Healthcare KGaA, Darmstadt, Germany; ^10^ Department of Medical Oncology, Centre Paul Strauss, Strasbourg, France

**Keywords:** bintrafusp alfa, triple-negative breast cancer, TGF-β, PD-L1, tumor microenvironment

## Abstract

**Background:**

We report the clinical activity, safety, and identification of a predictive biomarker for bintrafusp alfa, a first-in-class bifunctional fusion protein composed of the extracellular domain of TGFβRII (a TGF-β “trap”) fused to a human IgG1 mAb blocking PD-L1, in patients with advanced triple-negative breast cancer (TNBC).

**Methods:**

In this expansion cohort of a global phase 1 study, patients with pretreated, advanced TNBC received bintrafusp alfa 1200 mg every 2 weeks intravenously until disease progression, unacceptable toxicity, or withdrawal. The primary objective was confirmed best overall response by RECIST 1.1 assessed per independent review committee (IRC).

**Results:**

As of May 15, 2020, a total of 33 patients had received bintrafusp alfa, for a median of 6.0 (range, 2.0-48.1) weeks. The objective response rate was 9.1% (95% CI, 1.9%-24.3%) by IRC and investigator assessment. The median progression-free survival per IRC was 1.3 (95% CI, 1.2-1.4) months, and median overall survival was 7.7 (95% CI, 2.1-10.9) months. Twenty-five patients (75.8%) experienced treatment-related adverse events (TRAEs). Grade 3 TRAEs occurred in 5 patients (15.2%); no patients had a grade 4 TRAE. There was 1 treatment-related death (dyspnea, hemolysis, and thrombocytopenia in a patient with extensive disease at trial entry). Responses occurred independently of PD-L1 expression, and tumor RNAseq data identified *HMGA2* as a potential biomarker of response.

**Conclusions:**

Bintrafusp alfa showed clinical activity and manageable safety in patients with heavily pretreated advanced TNBC. *HMGA2 w*as identified as a potential predictive biomarker of response.

**ClinicalTrials.gov identifier:**

NCT02517398

## Introduction

Triple-negative breast cancer (TNBC) comprises 10% to 27% of breast cancers (1–5). TNBC is aggressive and associated with a poor prognosis, with an increased likelihood of distant recurrence and death within 5 years of diagnosis compared with other breast cancer subtypes (1–3). The 5-year US overall survival (OS) rate for women with advanced TNBC is only 12% (4). Currently, chemotherapy regimens with anthracyclines and taxanes are the standard first-line treatment for patients with TNBC (6, 7). While treatment with chemotherapy regimens elicits a complete response (CR) in up to 45% of patients with TNBC, those with residual disease after therapy have a greater likelihood of relapse or death compared with patients with other breast cancer subtypes (1, 8, 9). which may contribute to the poor prognosis of patients with TNBC overall.

PD-L1 signaling plays a key role in the immunosuppressive tumor microenvironment (TME) and is considered a predictive biomarker of response for anti–PD-(L)1 therapies in TNBC, where an estimated 20% of tumors express PD-L1 (10–13). In 2019, atezolizumab was approved in combination with nab-paclitaxel as a treatment option for PD-L1–positive (PD-L1–stained, tumor-infiltrating immune cells of any intensity covering ≥1% of the tumor area) metastatic TNBC; however, a follow-up study of atezolizumab plus paclitaxel failed to meet its primary endpoint of progression-free survival (PFS) for first-line treatment of patients with PD-L1–positive metastatic TNBC (14–16). In 2020, pembrolizumab in combination with chemotherapy was approved in the US based on improved median PFS in first-line treatment of patients with PD-L1–positive (combined positive score ≥10), metastatic TNBC (17).

Due to continued poor response rates with current standard-of-care therapies (10, 18, 19), new therapeutic options and novel predictive biomarkers of response to identify patients most likely to benefit from treatment remain significant unmet needs for patients with advanced TNBC.

The transforming growth factor β (TGF-β) pathway plays an important role in cancer progression and immune evasion by influencing the TME *via* regulatory effects on immune cells, and by promoting angiogenesis, fibrosis, and epithelial-mesenchymal transition (EMT) (20, 21). The TGF-β signaling pathway has been linked to worse outcomes in breast cancer, including disease progression, decreased relapse-free periods after surgical resection, shorter disease-free survival, and reduced OS (22–26). Therefore, inhibiting TGF-β activity in the TME while simultaneously blocking an additional immunosuppressive cellular mechanism, such as the PD-L1 pathway, may provide a new treatment approach for TNBC.

Bintrafusp alfa is a first-in-class bifunctional fusion protein composed of the extracellular domain of the human TGF-β receptor II (TGF-βRII or TGF-β “trap”) fused *via* a flexible linker to the C-terminus of each heavy chain of an IgG1 antibody blocking programmed death ligand 1 (anti–PD-L1) (27, 28). Bintrafusp alfa was designed to target tumors *via* colocalized, simultaneous blocking of 2 nonredundant immunosuppressive pathways (TGF-β and PD-L1) within the TME, and it specifically and efficiently depletes all 3 TGF-β isoforms (27). In 2 ongoing phase 1 studies (NCT02517398 and NCT02699515) in patients with heavily pretreated solid tumors, treatment with bintrafusp alfa has shown early signs of clinical activity and a manageable safety profile similar to that of anti–PD-(L)1 therapies (29, 30). Here, we report results from the global phase 1 study NCT02517398 evaluating bintrafusp alfa in an expansion cohort of patients with pretreated, advanced TNBC, including the results of an extensive, integrated tumor biomarker evaluation.

## Materials and methods

### Study design

This is a global, phase 1, open-label trial investigating the safety and clinical activity of bintrafusp alfa, including multiple expansion cohorts for patients with selected solid tumors. Eligible patients for the TNBC expansion cohort were ≥18 years old with confirmed TNBC that progressed during or after first-line therapy. Patients were required to have an Eastern Cooperative Oncology Group performance status (ECOG PS) of 0 or 1; life expectancy of ≥12 weeks; adequate renal, hepatic, and hematologic function; measurable disease by Response Evaluation Criteria in Solid Tumors (RECIST) version 1.1; and available archival tumor material or fresh biopsies taken within 28 days of the first administration of bintrafusp alfa. Prior treatment with immune checkpoint inhibitors, such as anti–PD-(L)1 or anti–CTLA-4 antibody, was not permitted. TNBC status was confirmed locally and was defined as <1% of tumor cells reactive to estrogen receptor and <1% of tumor cells reactive to progesterone receptor by immunohistochemistry (IHC), as well as one of the following: human epidermal growth factor receptor 2 (HER2) status of 0 or 1+ by IHC, 2+ by IHC *and* HER2 nonamplified negative by fluorescence *in situ* hybridization (FISH), or HER2 nonamplified negative by FISH. Patients received a flat dose of bintrafusp alfa 1200 mg as a 1-hour intravenous infusion every 2 weeks intravenously until confirmed progression, unacceptable toxicity, or trial withdrawal. To mitigate potential infusion-related reactions (IRRs), premedication with an antihistamine and acetaminophen 30-60 minutes prior to each dose of bintrafusp alfa was mandatory for the first 2 infusions and optional afterward. While changes in infusion rate and dose delays were allowed, dose reductions were not permitted.

Clinical activity was assessed by radiographic imaging 6 weeks after treatment initiation and every 6 weeks thereafter for the first year and then every 12 weeks. Tumor responses were assessed according to RECIST 1.1 and adjudicated by an independent end point review committee (IRC). Responses were confirmed by imaging at or more than 4 weeks from the first documentation of response, and progressive disease was confirmed by imaging between 4 and 6 weeks after progression had been diagnosed.

This trial was conducted in accordance with the ethical principles of the Declaration of Helsinki. Ethics committees at all participating institutions approved the trial protocol, and the trial was conducted in accordance with international standards of good clinical practice consistent with the International Conference on Harmonisation E6 Good Clinical Practice guideline. Each patient provided written informed consent prior to enrollment.

### Study endpoints

The primary endpoint was confirmed best overall response (BOR) as assessed by an IRC according to RECIST 1.1. Key secondary endpoints included investigator-assessed BOR and safety. Exploratory endpoints included PFS (ie, time from first administration of bintrafusp alfa until the first date of progressive disease (PD) or death due to any cause), duration of response (DOR), disease control rate (DCR) according to RECIST 1.1 as adjudicated by IRC, and OS (ie, time from first administration of bintrafusp alfa to the date of death due to any cause). Tumor response was assessed by radiographic imaging at baseline and every 6 weeks after initiating treatment for the first year and every 12 weeks thereafter. Adverse events (AEs) were assessed throughout treatment, for the first 28 days after the last study dose, at 10 weeks post treatment, and every 12 weeks thereafter. AE severity was assessed according to National Cancer Institute Common Terminology Criteria for Adverse Events version 4.03.

### Biomarker analyses

Exploratory analyses to identify potential predictive biomarkers for treatment response were also performed for this study. Sections from formalin-fixed, paraffin-embedded (FFPE) tumor tissue were assessed for PD-L1 expression and immune phenotype. RNA sequencing (RNAseq) was used to evaluate potential associations between biomarker expression and response to bintrafusp alfa treatment and to evaluate the association between gene expression and immune phenotype. Tumor cell and TME PD-L1 expression was assessed centrally with a proprietary assay, the rabbit monoclonal anti–PD–L1 antibody clone 73-10 (Dako), under license from Merck (31). Tumor cell PD-L1 expression was defined as positive or negative by a threshold level of ≥1% or <1% of PD-L1–positive tumor cells of any staining intensity, respectively. PD-L1 expression in TME was defined as percentage of tumor area covered by PD–L1-positive non-tumor cells at any staining intensity. PD–L1 expression in whole-tumor area was determined as the percentage of tumor area covered by PD-L1-positive tumor and non-tumor cells at any staining intensity (12). Tumor immune phenotype was determined using PD-L1 IHC, PD–L1 IHC–negative controls, and hematoxylin and eosin–stained sections, evaluated by a pathologist masked to the response data. “Inflamed” tumors were defined as tumors with lymphocytes in direct physical contact with tumor cells (28, 32). Tumors were considered “immune excluded” if ≥1% of the tumor stroma area was populated by lymphocytes. Immune cells could be located in the immediate vicinity of tumor cells but could not infiltrate tumor cell clusters. For these tumors, physical contact between lymphocytes and tumor cells was rare enough to be considered an exception rather than the rule (28, 32). Tumors were classified as “immune desert” if the tumor stroma area was populated only sparsely by lymphocytes (<1% of the tumor stroma area populated by lymphocytes), there were no dense immune cell infiltrates, and there was no contact of the immune cells with tumor cells (28, 32). RNAseq was performed on FFPE tissue samples for gene expression quantitation as previously described (28). The receiver operating characteristic (ROC) curve was evaluated with the confidence interval (CI) computed by R package pROC using default parameters (33).

### Statistical analyses

Planned enrollment for the TNBC cohort was 30 patients. With 30 patients treated, the study has approximately 87% power to rule out a ≤15% objective response rate (ORR; null hypothesis) when the true ORR is 35% at a 10% type I error rate (1-sided). The ORR was determined as the proportion of patients with a confirmed BOR of CR or partial response (PR). The uncertainty of estimates for the ORR was assessed by calculating a 95% exact (Clopper-Pearson) CI. The DCR was defined as the proportion of patients with a confirmed BOR of CR, PR, or stable disease (SD). DOR, PFS, and OS were analyzed using the Kaplan-Meier method. Descriptive statistics were used to analyze safety.

## Results

### Patient population and baseline characteristics

Between September 2016 and January 2017, a total of 51 patients were screened; 33 patients were enrolled in the TNBC expansion cohort and received ≥1 dose of bintrafusp alfa (**Supplementary Figure 1**). Median bintrafusp alfa treatment duration was 6 weeks (range, 2-48 weeks). The Kaplan-Meier analysis of follow-up time since first dose was 177 weeks (range, 4-178 weeks). At the time of data cutoff on May 15, 2020, no patients remained on treatment. Reasons for treatment discontinuation included progressive disease (PD; n=25), AEs (n=5), withdrawal of consent (n=1), protocol nonadherence (n=1), and other reason (disease progression and AE unrelated to bintrafusp alfa; n=1). Patient baseline characteristics are summarized in **Table 1**. This TNBC study population was heavily pretreated, with 29 patients (87.9%) having received prior radiotherapy and 18 patients (54.5%) having received ≥4 prior anticancer regimens. The majority of patients received taxanes (93.9%), anthracycline (78.8%), and platinum-based chemotherapy (66.7%) in prior regimens.

**Table 1:** Baseline patient and disease characteristics.

Characteristic	N = 33
Age, years	
Median (range)	49 (30-83)
Sex, n (%)	
Female	33 (100)
ECOG performance status, n (%)	
0	12 (36.4)
1	21 (63.6)
Location of metastases at baseline, n (%)	
Liver	14 (42.4)
Bone	9 (27.3)
Prior radiotherapy, n (%)	
Yes	29 (87.9)
No	4 (12.1)
No. of prior anticancer regimens, n (%)	
1	3 (9.1)
2	4 (12.1)
3	8 (24.2)
≥4	18 (54.5)
Prior treatment, n (%)	
Taxanes	31 (93.9)
Anthracycline	26 (78.8)
Platinum-based chemotherapy	22 (66.7)
Tumor cell PD-L1 expression, n (%)	
<1%	26 (78.8)
≥1%	4 (12.1)
Missing*	3 (9.1)

*Patients had insufficient number of tumor cells in biopsies for PD-L1 assessment. ECOG, Eastern Cooperative Oncology Group; PD-L1, programmed death ligand 1; TME, tumor microenvironment.

### Efficacy

**Figure 1:**
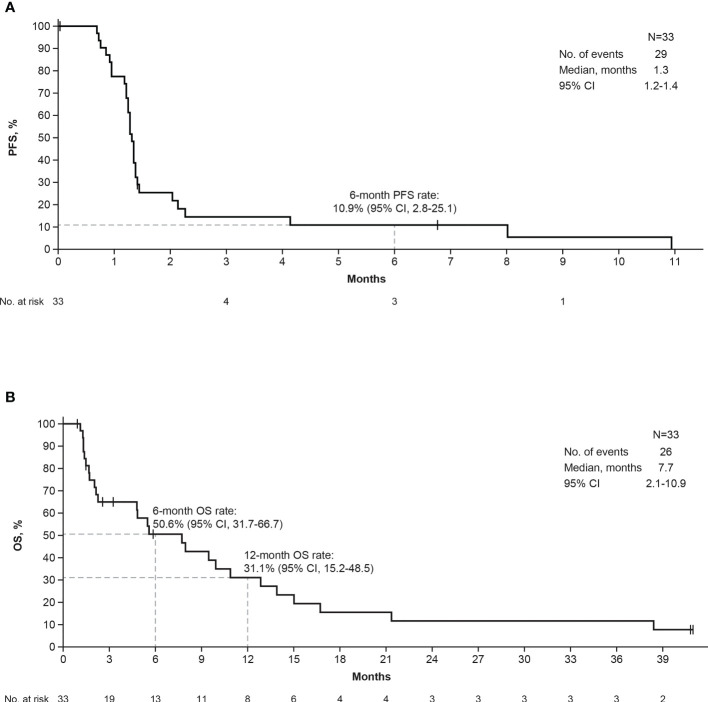
Kaplan-Meier curves of IRC-assessed PFS **(A)** and OS **(B)**. IRC, independent review committee; OS, overall survival; PFS, progression-free survival.

One patient had a confirmed CR and 2 patients had a confirmed PR per IRC assessment (ORR, 9.1%; 95% CI, 1.9%–24.3%); by investigator assessment, 3 patients had a confirmed PR (**Table 2**). The median DOR was 9.6 months (95% CI, 3.9-9.6 months) by IRC assessment and 5.4 months (95% CI, 5.3-5.5 months) by investigator assessment. The patient with a CR had a confirmed DOR of 9.6 months and discontinued bintrafusp alfa due to PD. The 2 patients with a PR had confirmed DORs of 5.5 and 3.9 months and discontinued bintrafusp alfa due to AEs and PD, respectively. For the patient who withdrew due to PD, treatment was initially interrupted due to multiple AEs (at which point the patient was assessed to have a PR by IRC and SD by investigator) and ultimately discontinued when PD was observed 3 months after the interruption. Two patients had SD ([DCR], 15.2%), with PFS of 1.4 and 4.1 months. No patients had an ongoing response at data cutoff. The percent change in target lesions over time by IRC assessment is shown in **Supplementary Figure 2**. The median PFS per IRC and investigator assessment was 1.3 months (95% CI, 1.2-1.4 months), with a 6-month PFS rate of 10.9% (95% CI, 2.8% to 25.1%) (**Figure 1A**). ORR, DCR, and median DOR were identical regardless of assessment per RECIST 1.1 or per iRECIST. The median OS was 7.7 months (95% CI, 2.1-10.9 months), with 7 patients alive at the time of cutoff (**Figure 1B**). The 6- and 12-month OS rates were 50.6% (95% CI, 31.7% to 66.7%) and 31.1% (95% CI, 15.2% to 48.5%), respectively.

**Table 2:** Confirmed overall responses according to RECIST 1.1.

Outcome	IRC assessed,	Investigator assessed,
	N = 33	N = 33
BOR, n (%)		
CR	1 (3.0)	0
PR	2 (6.1)	3 (9.1)
SD	2 (6.1)	2 (6.1)
PD	21 (63.6)	23 (69.7)
NE	7 (21.2)	5 (15.2)
ORR, n (%)	3 (9.1)	3 (9.1)
95% CI	1.9-24.3	1.9-24.3
DCR, n (%)	5 (15.2)	5 (15.2)
95% CI	5.1-31.9	5.1-31.9
Median DOR (range), months	9.6 (4.0-10.0)	5.4 (5.0-5.0)

BOR, best overall response; CR, complete response; DCR, disease control rate; DOR, duration of response; IRC, independent review committee; NE, not evaluable; PD, progressive disease; PR, partial response; ORR, objective response rate; RECIST, Response Evaluation Criteria in Solid Tumors; SD, stable disease.

### Safety

Twenty-five patients (75.8%) experienced treatment-related AEs (TRAEs) of any grade. The most common were diarrhea (18.2%; n=6), asthenia (15.2%; n=5), and anemia, headache, and nausea (12.1%; n=4 each) (**Table 3** **;** **Supplementary Table 1**). Grade 3 TRAEs occurred in 5 patients (15.2%). Three patients (9.1%) permanently discontinued treatment due to TRAEs: one patient due to grade 3 anemia; 1 patient due to grade 3 alanine aminotransferase (ALT) increased and aspartate aminotransferase (AST) increased; and 1 patient died due to dyspnea, hemolysis, and thrombocytopenia. For the patient who died, treatment discontinuation was due to grade 5 hemolysis, grade 5 dyspnea, and grade 2 breast hemorrhage. The death was assessed as treatment related by the investigator. This patient had extensive disease at trial entry and was noted to have multiple pulmonary emboli, PD, and expanding pleural effusion after 3 doses; no autoantibodies mediating hemolysis or thrombocytopenia were identified on workup. Immune-related AEs occurred in 4 patients (12.1%), including rash (6.1%; n=2) and ALT increased, AST increased, autoimmune thyroiditis, hypophysitis, and hypothyroidism (3.0%; n=1 each) (**Supplementary Table 2**). Two of these patients experienced multiple immune-related AEs (1 patient had ALT increased, AST increased, and rash; another patient had autoimmune thyroiditis and hypophysitis). IRRs that were related to treatment occurred in 4 patients (12.1%). Two patients (6.1%) had a grade 1 IRR, and 2 patients (6.1%) had a grade 2 IRR. Treatment-related skin lesions were reported in 2 patients (6.1%); each patient experienced both keratoacanthoma and squamous cell carcinoma of the skin (**Supplementary Table 2**), which resolved following shave biopsy and/or excision.

**Table 3:** TRAEs occurring at any grade in <10% of patients, TRAEs occurring at grade <3 and all AESIs.

N=33	Any grade	Grade 3	Grade 4	Grade 5
**Any TRAEs, n (%)**	25 (75.8)	5 (15.2)	0	1 (3.0)
Diarrhea	6 (18.2)	0	0	0
Asthenia	5 (15.2)	1 (3.0)	0	0
Anemia	4 (12.1)	3 (9.1)	0	0
Headache	4 (12.1)	0	0	0
Nausea	4 (12.1)	0	0	0
AST increased	3 (9.1)	1 (3.0)	0	0
ALT increased	2 (6.1)	1 (3.0)	0	0
Decreased appetite	1 (3.0)	1 (3.0)	0	0
Dyspnea	1 (3.0)	0	0	1 (3.0)*
Hemolysis	1 (3.0)	0	0	1 (3.0)*
Hypophysitis	1 (3.0)	1 (3.0)	0	0
Thrombocytopenia	1 (3.0)	0	0	1 (3.0)*
**Any AESI, n (%)**				
TGF-β inhibition–mediated skin AEs^†^	2 (6.1)	0	0	0
Immune-related AESIs	4 (12.1)	2 (6.1)	0	0
Immune-related rash	2 (6.1)	0	0	0
Immune related endocrinopathies: Thyroid disorders	2 (6.1)	0	0	0
Immune related endocrinopathies: Pituitary dysfunction	1 (3.0)	1 (3.0)	0	0
Immune-related hepatitis	1 (3.0)	1 (3.0)	0	0

*Dyspnea, hemolysis, and thrombocytopenia were reported as grade 5 TRAEs in the same patient. ^†^Includes MedDRA v23.0 preferred terms actinic keratosis, basal cell carcinoma, Bowen’s disease, hyperkeratosis, keratoacanthoma, lip squamous cell carcinoma, and squamous cell carcinoma of the skin. AESI, adverse event of special interest; ALT, alanine aminotransferase; AST, aspartate aminotransferase; MedDRA, Medical Dictionary for Regulatory Activities; TGF-β, transforming growth factor β; TRAE, treatment-related adverse event.

### Biomarker results

**Figure 2:**
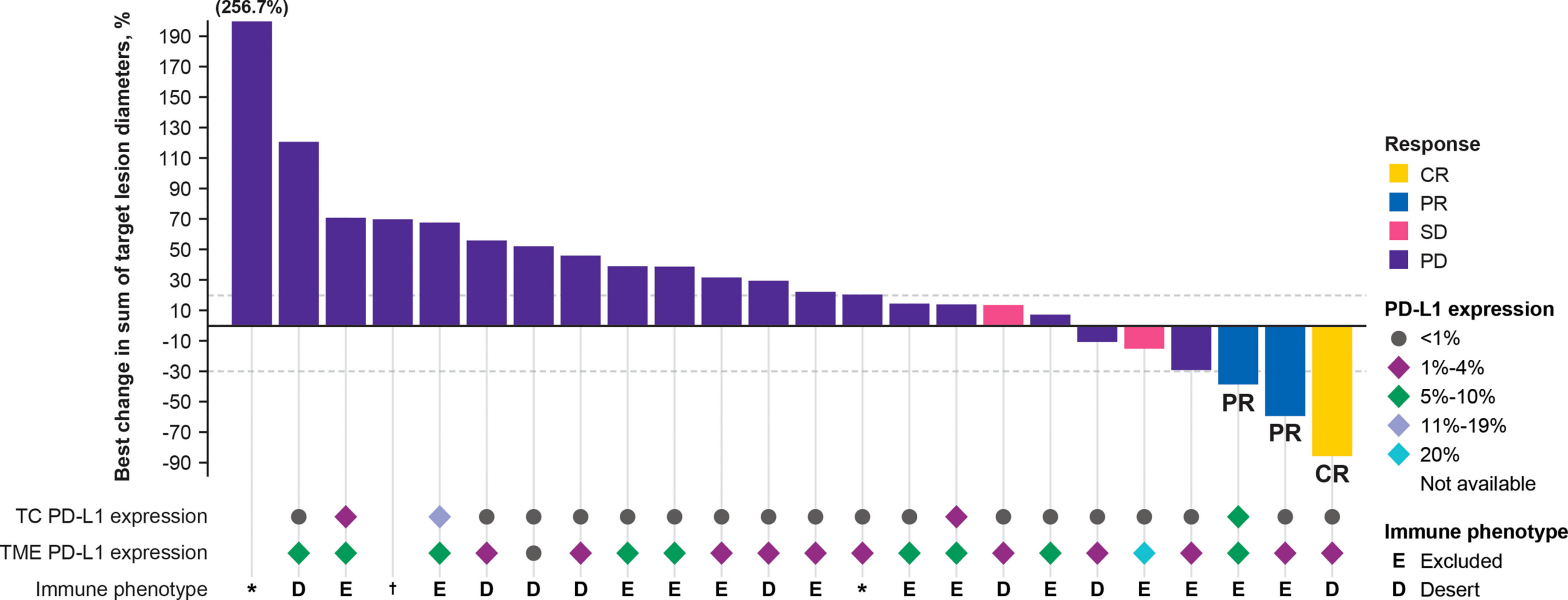
Best change in target lesions from baseline assessed by independent review committee. Patients with a BOR of NE (n = 7) are not included in this figure. Two additional patients with a BOR of PD were not included in this figure as they lacked a valid postbaseline target lesion measurement. *Non-evaluable immune phenotype. ^†^Sample not available for processing by pathologist. BOR, best overall response; CR, complete response; NE, not evaluable; PD, progressive disease; PD-L1, programmed death ligand 1; PR, partial response; SD, stable disease; TC, tumor cell; TME, tumor microenvironment.

**Figure 3:**
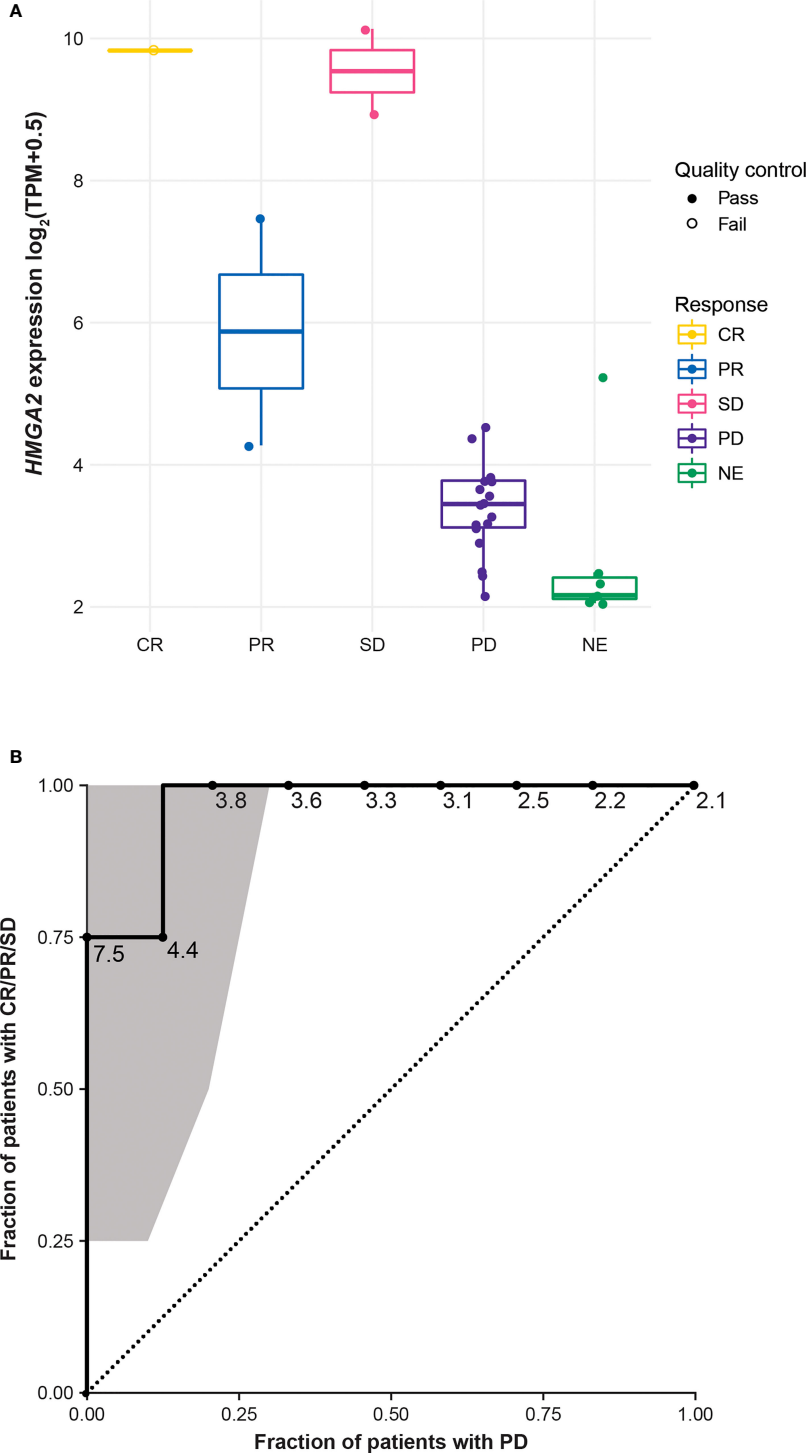
*HMGA2* expression by IRC-assessed BOR* **(A)** and ROC curve with the 95% CI computed by R package pROC using default parameters*^†^ **(B)**. *Three samples from patients with a BOR of PD are not included in this figure due to lack of sample or sequencing failure. ^†^One sample from a patient with a BOR of CR was excluded from the formal biomarker assessment due to failing quality control. BOR, best overall response; CR, complete response; HMGA2, high mobility group AT-hook 2; IRC, independent review committee; NE, not evaluable; PD, progressive disease; PR, partial response; ROC, receiver operating characteristic; SD, stable disease; TPM, transcripts per million.

Tumor cell and TME PD-L1 expression data were available in 30 of 33 patients. Among the 30 samples tested for PD-L1 expression, tumor type was invasive carcinoma of no special type in 29 (96.7%) and metaplastic carcinoma in 1 (3.3%). Eight of 30 samples (26.7%) were derived from core biopsies of the breast, 3 (10%) from needle biopsies of a liver metastasis, 10 (33.3%) from resections of the breast, 3 (10%) from resections of lymph node metastasis, 5 (16.7%) from resections of skin metastasis, and 1 (3.3%) from resection of lung metastasis. In patients with evaluable response and measurable lesion size, 20 of 24 samples (83.3%) of tumor cells did not express PD-L1, but 23 of 24 samples (95.8%) of the TME immune cells had PD-L1 expression ≥1% (**Figure 2**). TME PD-L1 expression reached 20% in 1 patient. Analysis of BOR and change in lesion size by PD-L1 expression and tumor immunophenotype showed that 2 of 3 responders (66.7%) had immune-excluded tumors and 1 responder (33.33%) had an immune-desert tumor (**Figure 2**). In all 30 patients in whom immunophenotype could be determined, an immune-desert phenotype was identified in 12/30 tissue samples (40%) and an immune-excluded phenotype in 18/30 tissue samples (60%). Two of 32 tissue samples had an indeterminate phenotype (6.3%), and no image was available for evaluation for 1 of 33 subjects (3.0%). Whole-transcriptome exploratory analysis of RNAseq data from tumor samples identified high expression of the high mobility group AT-hook 2 (*HMGA2*) gene as a potential predictive biomarker of response to bintrafusp alfa in TNBC. Three patients with a confirmed BOR of PD per IRC assessment did not have samples available for RNAseq. The sample from the single patient with a CR per IRC failed RNAseq quality control; this patient was excluded from differential expression analysis but was included in select analyses for full transparency. RNAseq samples passed quality control and were included in all analyses for patients with a confirmed BOR by IRC of PR (n=2), SD (n=2), PD (n=18), and not evaluable (NE; n=7). Tumor samples from 4 patients who experienced disease control (2 PRs and 2 SDs by IRC assessment) with bintrafusp alfa had a median 32-fold higher expression of *HMGA2* (q=4.34e-14 as computed by DESeq2) (34) than samples from patients who had PD (**Figure 3A**). The patient with a CR had a tumor with high *HMGA2* expression, despite the low data quality.

In patients with disease control on bintrafusp alfa treatment, the lowest *HMGA2* expression was 4.28 log_2_(transcripts per million [TPM] + 0.5). Using the 4.28 log_2_(TPM + 0.5) value as a cutoff, an additional 3 patients were considered to have high *HMGA2* expression; 2 patients had PD and 1 had a nonevaluable response (**Figure 3A**). Analysis of the *HMGA2* ROC curve identified very good accuracy of *HMGA2* in predicting disease control, with an area under the curve of 0.97 (**Figure 3B**), though this was not computed with an independent test set. Notably, *HMGA2* expression was found to be associated with response to bintrafusp alfa only in the TNBC cohort, and not in any other expansion cohorts in NCT02517398, including biliary tract cancer, non-small cell lung cancer, and squamous cell carcinoma of the head and neck (**Supplementary Figure 3**). Using the same cutoff for *HMGA2*-high expression, approximately 13.0% (23 of 177) of tumor samples annotated as TNBC in The Cancer Genome Atlas (TCGA) Breast Invasive Carcinoma data set (35) had high expression of *HMGA2* after adjustment with the ComBat algorithm (**Supplementary Figure 4**). *HMGA2* expression was not significantly associated with immune phenotype (modeling expression as a function of immune phenotype, using only n=27 samples with an immune phenotype call and valid RNAseq data, DESeq2 yields logFC=0.27, p=0.54, q=0.95).

## Discussion

Despite not meeting the primary endpoint, bintrafusp alfa demonstrated clinical activity and was well tolerated in this cohort of heavily pretreated patients with advanced TNBC. Disease control was achieved in 5 patients (15.2%), with an ORR of 9.1% per IRC assessment and a median DOR of 9.6 months; median PFS was 1.3 months and median OS was 7.7 months. The safety profile of bintrafusp alfa in this study was consistent with previously reported data on other cohorts (28, 30, 36, 37) and with what is expected for dual inhibition of TGF-β and PD-L1 (38). Grade 3 TRAEs occurred in 5 patients (15.2%), and there was 1 treatment-related death in a patient who had extensive disease at trial entry. Three patients (9.1%) permanently discontinued treatment due to TRAEs.

These efficacy data are consistent with historical data for patients with advanced TNBC who received second-line or later immune checkpoint inhibitor treatment. Studies with anti–PD-L1 inhibitors for second-line TNBC report PFS ranging from 1.4 to 2.1 months, OS ranging from 7.3 to 9.9 months, and ORR ranging from 5.2% to 9.6% (10, 18, 19, 39). In 2021, sacituzumab govitecan, an anti–Trop-2 antibody–drug conjugate, received US Food and Drug Administration approval for the treatment of patients with locally advanced or metastatic TNBC who have received ≥2 prior systemic therapies, including ≥1 therapy for metastatic disease (40). In a phase 1/2 trial, patients treated with sacituzumab govitecan for a mean of 9.6 cycles (range, 1-51 cycles) had an ORR by IRC of 34.3% (95% CI, 25.4% to 44.40%), a median DOR per IRC of 9.1 months (95% CI, 4.6-11.3 months), a median PFS per IRC of 5.5 months (95% CI, 4.1-6.3 months), and a median OS of 13.0 months (95% CI, 11.2-13.7 months) (41). In the subsequent phase 3 trial, treated patients had an ORR by IRC of 31%, a median DOR per IRC of 6.3 months (95% CI, 5.5-9.0 months), a median PFS per IRC of 4.8 months (95% CI, 4.1-5.8 months), and a median OS of 11.8 months (95% CI, 10.5-13.8 months) (42). TRAEs have been reported in 98%-100% of patients in sacituzumab govitecan trials, with neutropenia and diarrhea identified as the most clinically relevant AEs (41, 42). Despite the promising efficacy data, a predictive biomarker of response to sacituzumab govitecan has not been identified.

Analysis of the TME plays an important role in predicting response to chemotherapies and immunotherapies and clinical outcomes in patients with TNBC (43, 44). In our study, lymphocytic infiltration of tumors was low and restricted to the stroma (12 of 30 samples [40%] undergoing immunophenotypic analysis were designated immune desert, and 18 of 30 samples [60%] immune excluded), in line with the observation by Denkert et al. that the majority (70%) of TNBC samples had low or intermediate lymphocytic infiltration (43). A 10% increase in tumor-infiltrating lymphocytes has been associated with longer disease-free survival in patients with TNBC; increased lymphocytic infiltration has also been associated with longer OS (43). Therefore, the threshold of ≥1% or <1% of stromal lymphocytes that was used to distinguish between immune-excluded and immune-desert samples in this study might not be suitable for a profound analysis of the significance of lymphocytic infiltration in predicting response to, and clinical outcomes with, bintrafusp alfa treatment.

While we did not observe an association between response to bintrafusp alfa and PD-L1 expression on tumor cells and cells in the TME, RNAseq analysis of tumor samples from patients with TNBC identified high expression of the *HMGA2* gene to be a potential biomarker of response to bintrafusp alfa. The HMGA2 protein is a nonhistone architectural transcription factor that functions by altering chromatin structure and is involved in a variety of processes, including DNA repair, apoptosis, and senescence (45). HMGA2 is also an important factor in mediating TGF-β–induced EMT, a key event in cancer pathogenesis, and is upregulated by TGF-β/SMAD signaling (45, 46). In a TCGA data set (35), approximately 13.0% of patient samples annotated as TNBC had high *HMGA2* expression based on a ComBat adjusted cutoff of 0.72 log_2_(TPM + 0.5). While its expression in embryonic stem cells is critical during fetal development, *HMGA2* is not expressed or is only expressed at low levels in adult tissues (45). However, in tumors of mesenchymal and epithelial origin, high levels of HMGA2 protein expression are observed (45) and are associated with poorer OS in multiple types of cancer (47). While an analysis of 1,097 breast cancer samples in the TCGA data set did not identify *HMGA2* as a significant prognostic marker in breast cancer overall (47), its expression is predictive of relapse-free survival and metastasis in patients with TNBC (48). In preclinical studies, downregulation or inhibition of HMGA2 in TNBC tumors resulted in decreased metastasis (49, 50). In this manuscript, we report that expression of *HMGA2* in tumor samples from patients who experienced disease control was 32-fold higher than expression of *HMGA2* in samples from patients who had PD. The ORR for *HMGA2*-high tumors was 28.6% (2/7); the DCR was 57.1% (4/7). This is the first report of *HMGA2* as a potential predictive biomarker of response specifically for TNBC; however, given the small number of patients in this study and the exploratory nature of the analysis, larger studies are needed to validate this biomarker. Based on the link between TGF-β and HMGA2 activity in the literature and the efficacy and biomarker analyses presented here, TGF-β could present an important target in patients with TNBC and high *HMGA2* expression, and investigation into *HMGA2* as a potential predictive biomarker of response is warranted.

## Data availability statement

Any requests for data by qualified scientific and medical researchers for legitimate research purposes will be subject to Merck’s Data Sharing Policy. All requests should be submitted in writing to Merck’s data sharing portal (https://www.merckgroup.com/en/research/our-approach-to-research-and-development/healthcare/clinical-trials/commitment-responsible-data-sharing.html). When Merck has a co-research, co-development, or co-marketing or co-promotion agreement, or when the product has been out-licensed, the responsibility for disclosure might be dependent on the agreement between parties. Under these circumstances, Merck will endeavor to gain agreement to share data in response to requests.

## Ethics statement

This trial was conducted in accordance with the ethical principles of the Declaration of Helsinki. Ethics committees at all participating institutions approved the trial protocol, and the trial was conducted in accordance with international standards of good clinical practice consistent with the International Conference on Harmonisation E6 Good Clinical Practice guideline. The patients/participants provided their written informed consent to participate in this study.

## Author contributions

AS, LO, CH, PAR, ID, and GL contributed to conception of the study. LO and CH supervised the study. CH, PAR, CI, ID, and GL contributed to the development of the methodology. AS, AA, NI, DL, NP, and CB contributed to the provision of study resources. AS, AA, NI, DL, NP, CI, and CB contributed to the investigation process. LSO, CH, PAR, CI, ID, and GL contributed to data curation. AS, AA, NI, DL, NP, YZ, LO, CH, PAR, CI, ID, GL, and CB contributed to the formal analysis. AS, AA, NI, DL, NP, YZ, LO, CH, PAR, CI, ID, GL, and CB participated in writing the original draft of the manuscript. AS, AA, NI, DL, NP, YZ, LO, CH, PAR, CI, ID, GL, and CB participated in reviewing and revising the manuscript. All authors contributed to the article and approved the submitted version.
