# Supplementary material for: Identification of HMGA2 as a predictive biomarker of response to bintrafusp alfa in a phase 1 trial in patients with advanced triple-negative breast cancer
Source: Front Oncol. 2022 Dec 8;12:981940. doi: 10.3389/fonc.2022.981940 (PMC9773992; doi:10.3389/fonc.2022.981940)
Supplement: Supplementary file 1 [file DataSheet_1.docx]

Supplementary Material


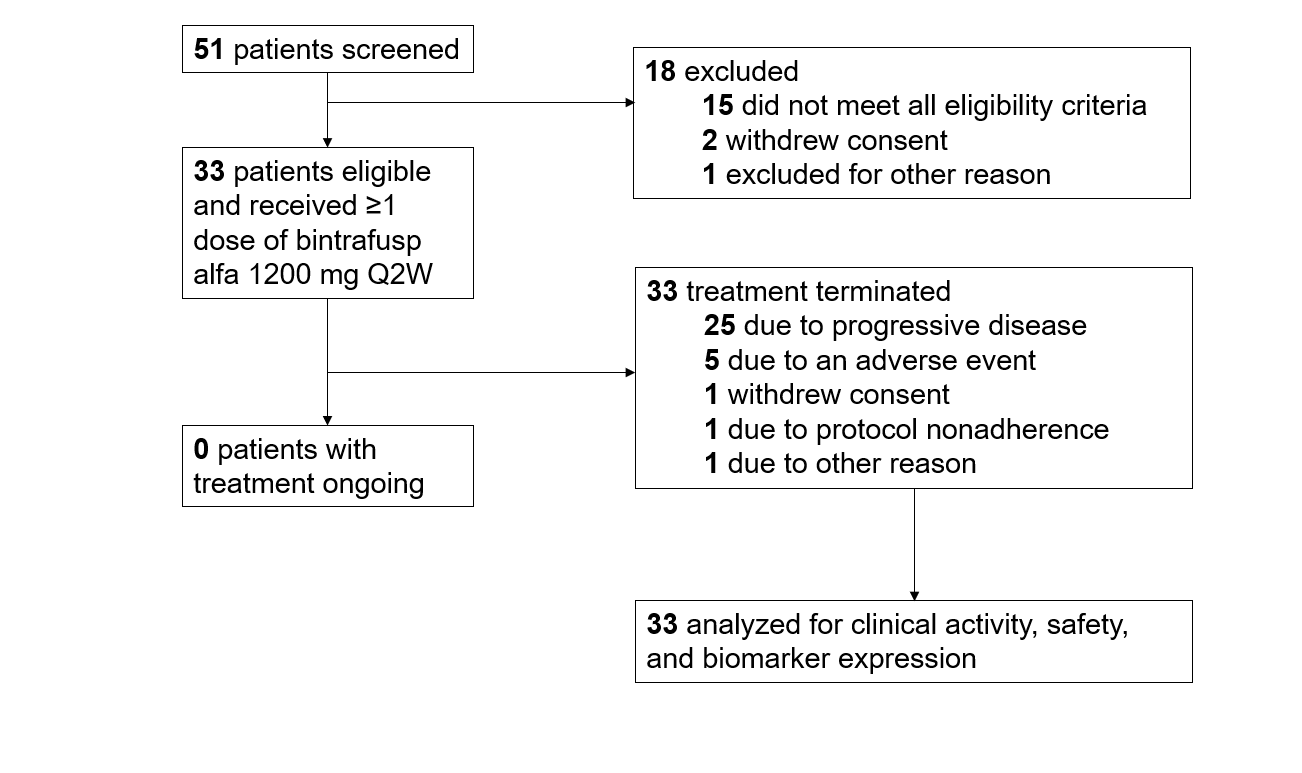


**Supplementary Figure 1.** Trial profile. Q2W, every 2 weeks.


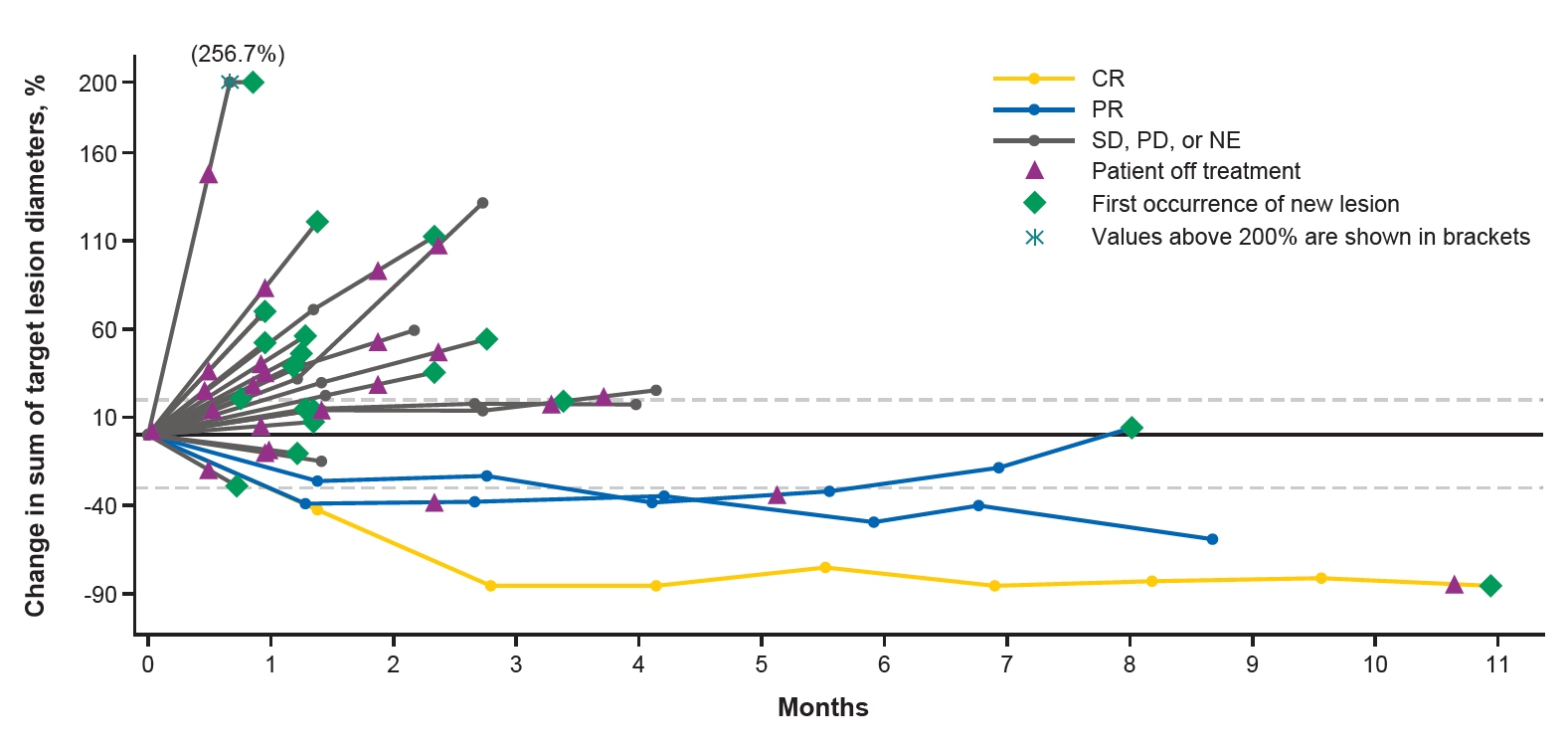


**Supplementary Figure 2.** Percent change from baseline in target lesion diameter over time as assessed by IRC. Patients with a BOR of NE (n=7) are not included in this figure. Five additional patients were not included because they lacked a valid postbaseline target lesion measurement. BOR, best overall response; CR, complete response; IRC, independent review committee; NE, not evaluable; PD, progressive disease; PR, partial response; SD, stable disease.


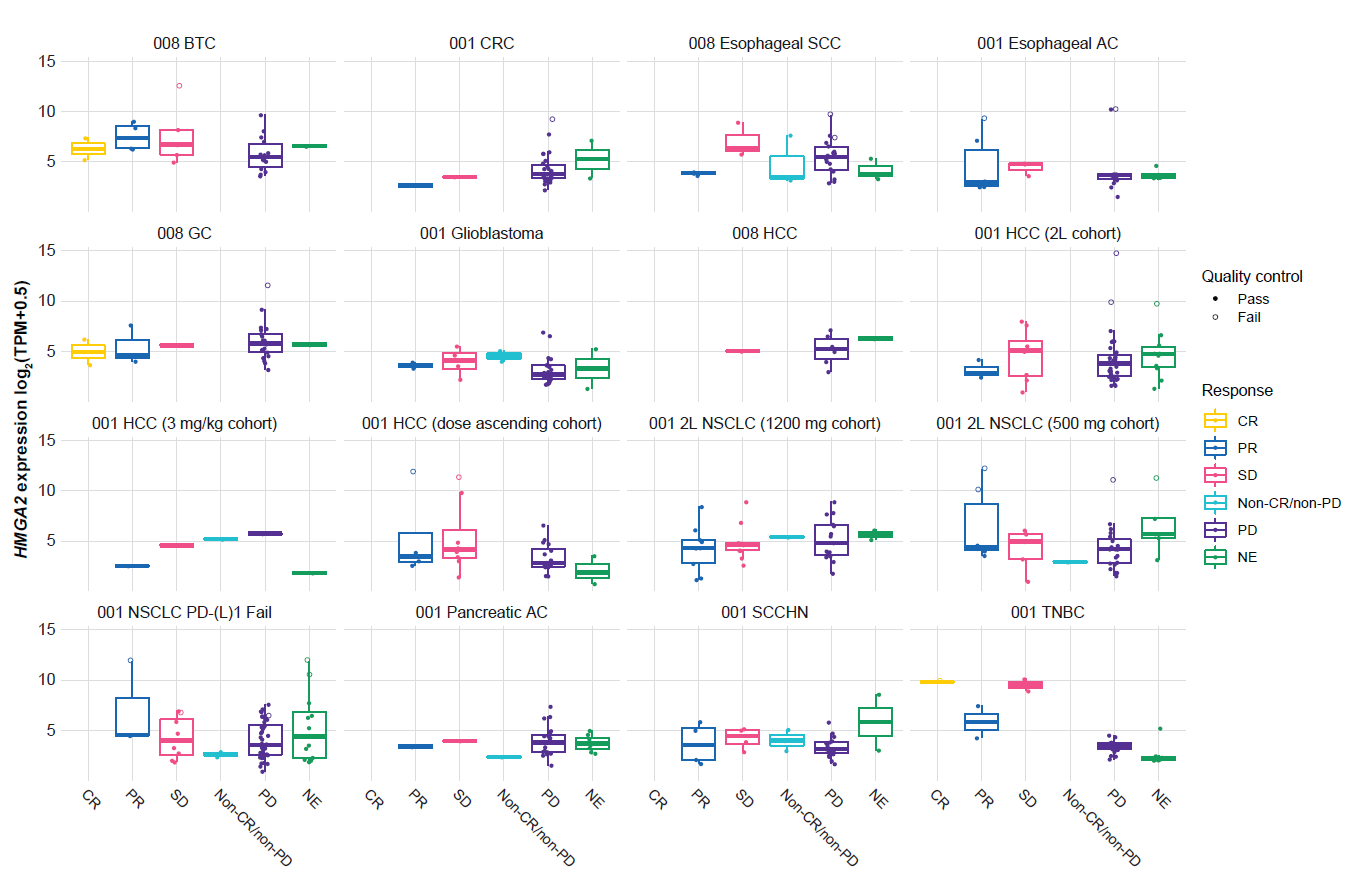


**Supplementary Figure 3.** *HMGA2* expression level and confirmed BOR by IRC in solid tumor cohorts. 2L, second line; AC, adenocarcinoma; BOR, best overall response; BTC, biliary tract cancer; CR, complete response; CRC, colorectal cancer; ESCC, esophageal squamous cell carcinoma; GC, gastric cancer; HCC, hepatocellular carcinoma; HMGA2, high mobility group AT-hook 2; IRC, independent review committee; NE, not evaluable; NSCLC, non-small cell lung cancer; PD, progressive disease; PD-(L)1, programmed death ligand 1; PR, progressive disease; SCC, squamous cell carcinoma; SCCHN, squamous cell carcinoma of the head and neck; SD, stable disease; TNBC, triple-negative breast cancer; TPM, transcripts per million.


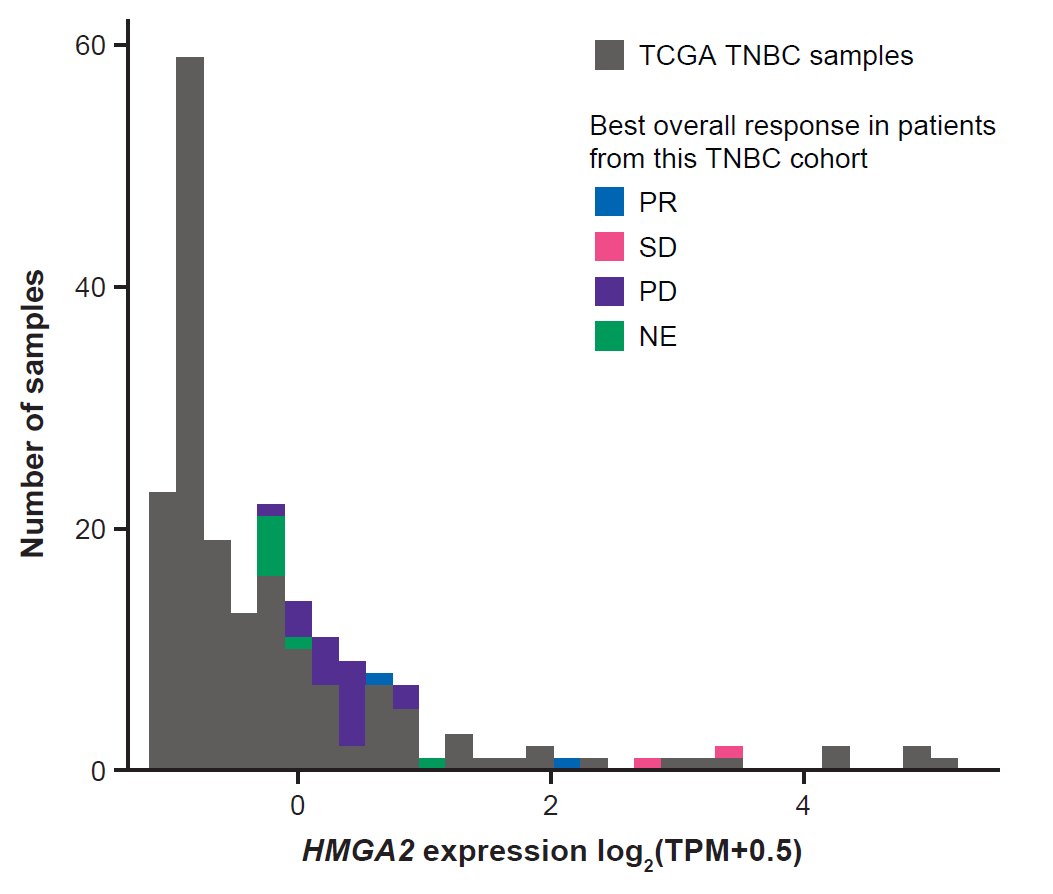


**Supplementary Figure 4.** Distribution of *HMGA2* expression* in TNBC samples from NCT02517398 and TCGA data set.^†‡^ *The ComBat-adjusted cutoff for *HMGA2*-high expression is 0.72 log_2_(TPM+0.5). ^†^TNBC samples identified from the Breast Invasive Carcinoma TCGA data set. ^‡^One sample from a patient with a BOR of CR is not included in this figure due to failing quality control. Three samples from patients with a BOR of PD are not included in this figure due to lack of sample or sequencing failure. BOR, best overall response; CR, complete response; HMGA2, high mobility group AT-hook 2; NE, not evaluable; PD, progressive disease; PR, partial response; SD, stable disease; TCGA, The Cancer Genome Atlas; TNBC, triple-negative breast cancer; TPM, transcripts per million.

**Supplementary Table 1.** TRAEs occurring at any grade and of grade ≥3. TRAE, treatment-related adverse event.

| **N=33** | **Any grade** | **Grade ≥ 3** |
| --- | --- | --- |
| **TRAEs, n (%)** |  |  |
| Anemia | 4 (12.1) | 3 (9.1) |
| Eosinophilia | 1 (3.0) | 0 |
| Hemolysis | 1 (3.0) | 1 (3.0) |
| Thrombocytopenia | 1 (3.0) | 1 (3.0) |
| Autoimmune thyroiditis | 1 (3.0) | 0 |
| Hypophysitis | 1 (3.0) | 1 (3.0) |
| Hypothyroidism | 1 (3.0) | 0 |
| Colitis | 1 (3.0) | 0 |
| Constipation | 1 (3.0) | 0 |
| Diarrhea | 6 (18.2) | 0 |
| Dry mouth | 1 (3.0) | 0 |
| Dyspepsia | 1 (3.0) | 0 |
| Gingival bleeding | 1 (3.0) | 0 |
| Nausea | 4 (12.1) | 0 |
| Oral pain | 1 (3.0) | 0 |
| Vomiting | 1 (3.0) | 0 |
| Asthenia | 5 (15.2) | 1 (3.0) |
| Fatigue | 1 (3.0) | 0 |
| Pyrexia | 3 (9.1) | 0 |
| Xerosis | 1 (3.0) | 0 |
| Tonsillitis | 1 (3.0) | 0 |
| Infusion related reaction | 2 (6.1) | 0 |
| Alanine aminotransferase  increased | 2 (6.1) | 1 (3.0) |
| Aspartate aminotransferase  increased | 3 (9.1) | 1 (3.0) |
| Blood bilirubin increased | 1 (3.0) | 0 |
| Decreased appetite | 1 (3.0) | 1 (3.0) |
| Hypoalbuminemia | 1 (3.0) | 0 |
| Hypokalemia | 1 (3.0) | 0 |
| Arthralgia | 1 (3.0) | 0 |
| Muscular weakness | 1 (3.0) | 0 |
| Myalgia | 1 (3.0) | 0 |
| Keratoacanthoma | 2 (6.1) | 0 |
| Squamous cell carcinoma of skin | 2 (6.1) | 0 |
| Headache | 4 (12.1) | 0 |
| Breast hemorrhage | 1 (3.0) | 0 |
| Dyspnea | 1 (3.0) | 1 (3.0) |
| Epistaxis | 2 (6.1) | 0 |
| Pneumonitis | 1 (3.0) | 0 |
| Dry skin | 2 (6.1) | 0 |
| Erythema | 1 (3.0) | 0 |
| Papule | 1 (3.0) | 0 |
| Pruritus | 3 (9.1) | 0 |
| Purpura | 1 (3.0) | 0 |
| Rash | 3 (9.1) | 0 |
| Rash maculo-papular | 1 (3.0) | 0 |
| Rash papular | 1 (3.0) | 0 |
| Skin hemorrhage | 1 (3.0) | 0 |
| Telangiectasia | 1 (3.0) | 0 |

**Supplementary Table 2.** Patients reporting AEs of special interest. * Includes actinic keratosis, basal cell carcinoma, Bowen’s disease, hyperkeratosis, keratoacanthoma, lip squamous cell carcinoma, and squamous cell carcinoma of the skin MedDRA v23.0 preferred terms. AE, adverse event.

| **N=33** | **Any grade** | **Grade 3** |
| --- | --- | --- |
| **Any immune-related AE, n (%)** | 4 (12.1) | 2 (6.1) |
| Immune-related rash | 2 (6.1) | 0 |
| Rash | 2 (6.1) | 0 |
| **Immune-related endocrinopathies: thyroid disorders** | 2 (6.1) | 0 |
| Autoimmune thyroiditis | 1 (3.0) | 0 |
| Hypothyroidism | 1 (3.0) | 0 |
| **Immune-related hepatitis** | 1 (3.0) | 1 (3.0) |
| Alanine aminotransferase level increased | 1 (3.0) | 1 (3.0) |
| Aspartate aminotransferase level increased | 1 (3.0) | 1 (3.0) |
| **Immune related endocrinopathies: pituitary dysfunction** | 1 (3.0) | 1 (3.0) |
| Hypophysitis | 1 (3.0) | 1 (3.0) |
| **TGF-β inhibition–mediated skin adverse events*** | 2 (6.1) | 0 |
| Keratoacanthoma | 2 (6.1) | 0 |
| Squamous cell carcinoma of skin | 2 (6.1) | 0 |
